# Modulation of corticospinal excitability related to the forearm muscle during robot-assisted stepping in humans

**DOI:** 10.1007/s00221-023-06565-1

**Published:** 2023-03-17

**Authors:** Taku Kitamura, Yohei Masugi, Shin-ichiroh Yamamoto, Toru Ogata, Noritaka Kawashima, Kimitaka Nakazawa

**Affiliations:** 1 Department of Bio-Science and Engineering, Graduate School of Engineering and Science, Shibaura Institute of Technology, Saitama-shi, Saitama Japan; 2 Motor Control Section, Department of Rehabilitation for Movement Functions, Research Institute, National Rehabilitation Center for Persons with Disabilities, Tokorozawa-shi, Saitama Japan; 3 Robotics Program, Tokyo Metropolitan College of Industrial Technology, Arakawa-ku, Tokyo Japan; 4 Department of Life Sciences, Graduate School of Arts and Sciences, The University of Tokyo, 3-8-1 Komaba, Meguro-Ku, Tokyo, 153-8902 Japan; 5 Department of Physical Therapy, School of Health Sciences, Tokyo International University, Kawagoe-shi, Saitama Japan; 6 Department of Bio-Science and Engineering, College of Systems Engineering and Science, Shibaura Institute of Technology, Saitama-shi, Saitama Japan; 7 Department of Rehabilitation Medicine, The University of Tokyo Hospital, Bunkyo-ku, Tokyo Japan

**Keywords:** Lokomat, Robotic gait orthosis, Transcranial magnetic stimulation, H-reflex, Somatosensory input

## Abstract

In recent years, the neural control mechanisms of the arms and legs during human bipedal walking have been clarified. Rhythmic leg stepping leads to suppression of monosynaptic reflex excitability in forearm muscles. However, it is unknown whether and how corticospinal excitability of the forearm muscle is modulated during leg stepping. The purpose of the present study was to investigate the excitability of the corticospinal tract in the forearm muscle during passive and voluntary stepping. To compare the neural effects on corticospinal excitability to those on monosynaptic reflex excitability, the present study also assessed the excitability of the H-reflex in the forearm muscle during both types of stepping. A robotic gait orthosis was used to produce leg stepping movements similar to those of normal walking. Motor evoked potentials (MEPs) and H-reflexes were evoked in the flexor carpi radialis (FCR) muscle during passive and voluntary stepping. The results showed that FCR MEP amplitudes were significantly enhanced during the mid-stance and terminal-swing phases of voluntary stepping, while there was no significant difference between the phases during passive stepping. Conversely, the FCR H-reflex was suppressed during both voluntary and passive stepping, compared to the standing condition. The present results demonstrated that voluntary commands to leg muscles, combined with somatosensory inputs, may facilitate corticospinal excitability in the forearm muscle, and that somatosensory inputs during walking play a major role in monosynaptic reflex suppression in forearm muscle.

## Introduction

Human bipedal walking is thought to share common locomotor neural substrates with quadrupedal mammalian walking (Dietz 2002). Several studies have demonstrated that neural connections between the arms and legs are preserved in the human central nervous system (Dietz 2002; Huang and Ferris 2004; Zehr and Duysens 2004; Kawashima et al. 2008). For example, arm cycling movements that mimic locomotion modulate excitability in both the corticospinal tract and the monosynaptic reflex circuits of leg muscles (de Ruiter et al. 2010; Frigon et al. 2004), while leg cycling modulates the excitability of these circuits in arm muscles (Zehr et al. 2007). These results indicate that neural interactions occur between the arms and legs at the cortical and subcortical levels. It follows that both somatosensory inputs and central drive from the motor cortex or subcortical regions cause these modulations during locomotor-like activity. These results provide valuable information regarding neural systems related to human walking. However, the cycling movements used in these studies had different characteristics from those of natural walking. For example, cycling was performed in a sitting position. In addition, cycling has different loading and movement patterns compared to walking. Several studies have reported posture-dependent reflex modulation (Angulo-Kinzler et al. 1998). Others have reported that load-related sensory input alters corticospinal excitability during walking (Kamibayashi et al. 2009). These factors could potentially cause a difference in neural activity. Therefore, it is unclear whether these findings using the cycling paradigm can be applied to bipedal walking.

Recently, the driven gait orthosis system (Lokomat, Hocoma AG, Zurich, Switzerland) has been used to examine the effects of somatosensory inputs on the human central nervous system (Nakajima et al. 2008, 2011, 2016; Kamibayashi et al. 2010; Masugi et al. 2015). This system simulated stepping movements without voluntary effort in participants with an upright posture. These movements are more similar to walking than cycling. In addition, the system provides foot contact during the stance phase. Previous studies have reported that the excitability of the monosynaptic reflex in forearm muscles is suppressed during both voluntary stepping (Domingo et al. 2014) and passive stepping (Nakajima et al. 2011). Somatosensory inputs from the legs during walking may enhance the presynaptic inhibition of Ia terminals in the monosynaptic reflex circuits of the forearm muscles (Frigon et al. 2004; Nakajima et al. 2016). However, although these studies showed interlimb interactions in monosynaptic reflex circuits, it remains unclear whether and how excitability of the corticospinal tract is modulated in forearm muscles during human walking.

The corticospinal tract is partially involved in the control of the arms during human walking. Previous animal studies have shown that the motor cortex and corticospinal tract play an important role in the neural control of locomotion (Armstrong and Drew 1985; Drew 1993). In human studies, supraspinal control mechanisms of human walking have been investigated using transcranial magnetic stimulation (TMS), a non-invasive brain stimulation method (Schubert et al. 1997; Capaday et al. 1999; Kamibayashi et al. 2009). Using this technique, Barthelemy and Nielsen (2010) showed that the corticospinal tract contributes to arm muscle activity during walking. However, the extent to which afferent information induced by walking contributes to the modulation of corticospinal excitability in forearm muscles remains unclear. Addressing this question will bring us closer to understanding the neural mechanism of interlimb coordination during walking.

The purpose of the present study was to investigate excitability in the corticospinal tract and monosynaptic reflex circuits of the forearm muscles during both passive and voluntary stepping. Based on the findings of previous studies (Barthelemy and Nielsen, 2010; Nakajima et al. 2011; Domingo et al. 2014), we hypothesized that (1) the excitability of the corticospinal tract would change in a phase-dependent manner during voluntary stepping, but not during passive stepping, and (2) the excitability of the monosynaptic reflex would be suppressed during both voluntary and passive stepping.

## Materials and methods

### Participants

Fourteen healthy men with no history of neurological disorders participated in this study (Table 1). Three experiments were performed on different days. In Experiment 1, corticospinal excitability in the forearm muscle (flexor carpi radialis; FCR) was examined during voluntary stepping and passive standing. In Experiment 2, corticospinal excitability in the FCR was investigated during passive stepping and passive standing. In Experiment 3, monosynaptic reflex excitability was examined during voluntary stepping, passive stepping, and passive standing. The experimental protocols were approved by the local ethics committee of the National Rehabilitation Center for Persons with Disabilities (Saitama, Japan) and were in accordance with the guidelines set out in the Declaration of Helsinki (1964). Informed consent was obtained from all participants included in this study.

**Table 1:** Demographic data of the participants in the three experiments

Participant	Sex	Age	Experiment 1 MEP Voluntary stepping	Experiment 2 MEP Passive stepping	Experiment 3 H-reflex Voluntary stepping Passive stepping
ID		(years)	*n* = 9	*n* = 9	*n* = 9
1	male	23	〇	〇	〇
2	male	24	〇	〇	〇
3	male	24	〇	×	×
4	male	24	〇	×	×
5	male	21	〇	〇	〇
6	male	32	〇	〇	×
7	male	26	〇	〇	〇
8	male	22	〇	〇	×
9	male	31	〇	〇	×
10	male	22	×	〇	〇
11	male	22	×	〇	〇
12	male	22	×	×	〇
13	male	33	×	×	〇
14	male	29	×	×	〇

### General procedures

**Fig. 1:**
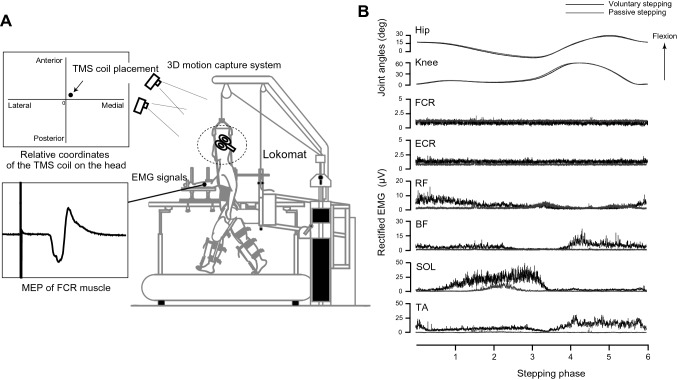
Experimental setup of Experiments 1 and 2 (**A**) Leg stepping movements were controlled by a driven gait orthosis (Lokomat). Motor evoked potentials were recorded from the right flexor carpi radialis (FCR). To keep the transcranial magnetic stimulation coil in place, a custom-made coil navigation system was used. **B** Typical examples of right hip and knee joint angles and electromyographic (EMG) activities in the right FCR, extensor carpi radialis (ECR), rectus femoris (RF), biceps femoris (BF), soleus (SOL), and tibialis anterior (TA). These data were obtained from a single subject during passive and voluntary stepping. EMG signals of fifteen gait cycles were full-wave rectified and averaged across gait cycles

In all three experiments, the Lokomat system was used to move the legs of the participants along predetermined walking trajectories with 40% body weight support (BWS). Detailed information on the Lokomat can be found elsewhere (Colombo et al. 2000). Briefly, the Lokomat system consists of a treadmill, a BWS system, and two leg orthoses that can move each leg (Fig. 1A). Lokomat can simulate human walking (i.e., upright posture, cyclic leg movements, and foot contact). It enables researchers to investigate the effects of somatosensory inputs on the human central nervous system during walking (Kamibayashi et al. 2010; Nakajima et al. 2011; Domingo et al. 2014).

The participants were fixed into the Lokomat using a harness to support their body weight, with cuffs around the upper and lower thighs of both legs. To prevent their right forearm from moving during the experiments, their right forearm, wrist, and hand were fixed to a rigid platform, with the elbow flexed at 90° and the wrist in the neutral position. The left forearm, wrist, and hand were not fixed. The treadmill speed was set to 2.0 km/h, and the range of hip and knee joint motion was set to 45° and 60°, respectively. This system provides analog signals of the hip and knee joint angles of both legs.

The present study included the following three tasks using the Lokomat system: (1) voluntary stepping, (2) passive stepping, and (3) passive standing. In both stepping tasks, the system produced the same movement of the hip and knee joints with 40% body weight support. For passive stepping, participants were asked to relax their body during stepping and not interfere with the movements of the robotic orthoses. Dorsiflexion of the ankle joint during passive stepping tasks was achieved using passive foot lifters (spring-assisted elastic straps) to prevent foot drop during the swing phase (Kamibayashi et al. 2010). For voluntary stepping, participants were asked to move their legs in response to the movement of the robotic orthoses. Foot lifters were not used in the voluntary stepping task to allow the participants to activate their lower leg muscles during stepping. For passive standing, the participants were fixed into the Lokomat system with 40% body weight support and asked to relax their body during passive standing. To prevent knee bending, the participant’s legs were supported by an experimenter. During the measurement of these three tasks, the participants were asked to relax both arms. Excitability of the corticospinal tract and the monosynaptic reflex in the forearm muscle was assessed during these three tasks.

### Corticospinal excitability (Experiment 1 and Experiment 2)

In Experiments 1 and 2, corticospinal excitability in the forearm muscle was investigated using TMS. Figure 1A shows the setup of Experiments 1 and 2, including the TMS, custom-made TMS coil navigation system, and the Lokomat system. To induce motor evoked potentials (MEPs) in the right FCR muscle, single-pulse TMS was applied to the left primary motor cortex using a figure-eight coil (90-mm diameter) connected to a magnetic stimulator (Magstim200; Magstim Co., Whitland, UK). The coil was placed tangentially on the scalp, with the handle pointing backward and laterally at 45° from the midline. The optimal coil location (the “hotspot”) was determined as the location at which the largest MEPs were observed in the right FCR during the passive standing task. Next, we determined the resting motor threshold (rMT), which was defined as the minimum stimulus intensity to produce FCR MEP amplitudes of at least 50 µV in five out of 10 consecutive trials. Stimulus intensity was set to 1.1 times the rMT of the FCR MEP. The stimulus intensity and coil location were maintained throughout the experiment. The inter-stimulus interval was set to > 8 s.

In general, it would be difficult for an experimenter to keep the TMS coil positioned on the hotspot while the participant conducted the stepping tasks. Therefore, different parts of the motor cortex are likely activated by TMS during stepping. To solve this problem, we used a custom-made TMS coil navigation system. Detailed information can be found in our previous report (Kitamura et al. 2013). Briefly, the system consists of a 3D motion capture system (OptiTrack V100:R2; Natural Point Inc., Oregon, USA) and a custom-written LabVIEW program (National Instruments, Texas, USA). The system can provide real-time visual feedback to the experimenters regarding the relative coordinates of the stimulus coil on the head of the participants. Using this system, the experimenter kept the TMS coil location constant throughout the experiment.

In Experiment 1, corticospinal excitability in the right FCR muscle was investigated during voluntary stepping and passive standing. TMS was applied in six phases during stepping. In voluntary stepping, one step cycle was fixed to 2.16 s. TMS was pseudo-randomly delivered at 360 (Phase 1), 720 (Phase 2), 1080 (Phase 3), 1440 (Phase 4), 1800 (Phase 5), and 2160 ms (Phase 6) after maximum extension of the left hip joint (Fig. 1B). Phases 1–3 represent the stance phase of the right leg and Phases 4–6 represent the swing phase of the right leg. First, 15 MEPs were obtained during passive standing. Second, 15 MEPs were obtained during each phase of voluntary stepping. As a practice for voluntary stepping, the participants practiced for approximately five minutes before starting the measurement. Finally, EMG signals were recorded during maximum voluntary contraction (MVC). For the forearm muscles, the activity during maximal isometric contraction was measured using manual resistance while being fixed to the Lokomat. For the leg muscles, the Lokomat was removed, and the EMG activity during maximal isometric contraction was measured using manual resistance.

In Experiment 2, corticospinal excitability in the right FCR was investigated during passive stepping and passive standing. TMS was applied in six phases during stepping. In passive stepping, one step cycle was fixed to 2.16 s. TMS was pseudo-randomly delivered at 360 (Phase 1), 720 (Phase 2), 1080 (Phase 3), 1440 (Phase 4), 1800 (Phase 5), and 2160 ms (Phase 6) after maximum extension of the left hip joint (Fig. 1B). Phases 1–3 represent the stance phase of the right leg and Phases 4–6 represent the swing phase of the right leg. First, 15 MEPs were obtained during passive standing. Second, 15 MEPs were obtained during each phase of passive stepping. As a practice for passive stepping, the participants practiced for approximately five minutes before starting the measurement. Finally, EMG signals were recorded during MVC.

**Fig. 2:**
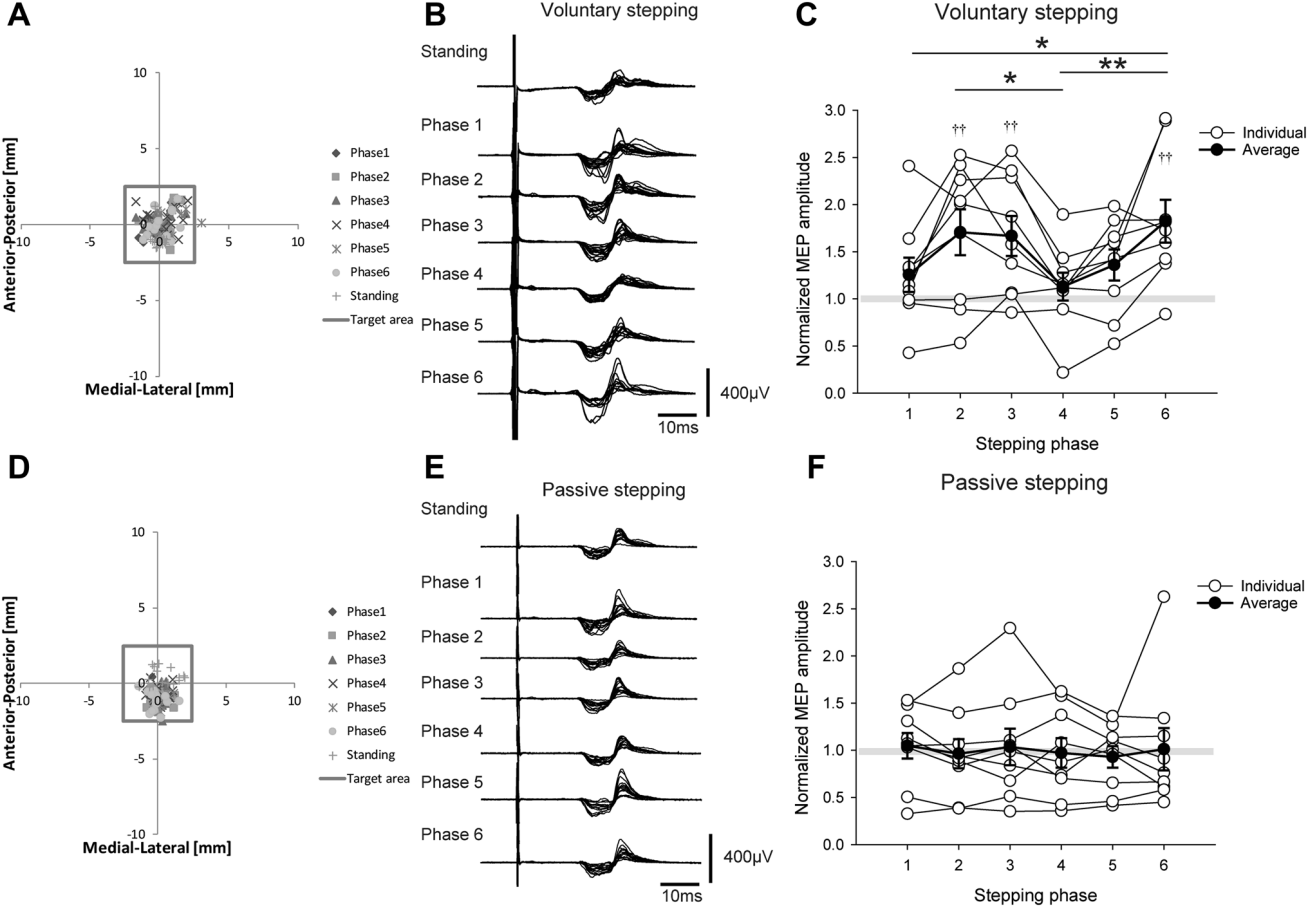
Motor evoked potentials (MEPs) obtained during voluntary stepping and passive stepping tasks. The scatter plots in **A** and **D** show coil location when transcranial magnetic stimulation (TMS) was delivered during voluntary stepping and passive stepping in a single subject, respectively. The results in the passive standing condition are also overlaid in Fig. 2A and 2D. The square area indicates the target area for TMS around a hotspot. Typical MEP waveforms (15 sweeps) obtained from the flexor carpi radialis (FCR) muscle during voluntary stepping (**B**) and passive stepping (**E**). These typical examples (**A**, **B**, **D**, and **E**) were obtained from the same subject on different days. (**C**) and (**F**) The averages (± standard error) of the MEP amplitudes in the FCR muscle obtained from nine subjects during voluntary stepping (**C**) and passive stepping (**F**). MEP amplitudes were normalized by the amplitudes of the MEP recorded during the standing condition (*p < 0.05; **p < 0.01 for difference between the stepping phases; †*p* < 0.05; ††*p* < 0.01 for difference between each stepping phase and passive standing)

Figure 2A, D shows typical examples (*n* = 1) of TMS coil location data during voluntary and passive stepping, respectively. If the TMS coil was placed outside the 5 × 5 mm square, the MEP data were excluded from the analysis.

### Monosynaptic reflex (Experiment* 3)*

In Experiment 3, the excitability of the Hoffmann reflex (H-reflex) pathway of the FCR was investigated during voluntary stepping, passive stepping, and passive standing. H-reflexes were evoked in the right FCR muscle using a constant-current electrical stimulator (SEN-7023; Nihon Kohden, Tokyo, Japan). To stimulate the right median nerve, rectangular pulse electrical stimulations (1-ms duration) was delivered just proximal to the medial epicondyle of the humerus (Zehr et al. 2007; Nakajima et al. 2011). Electrical stimuli were delivered at six different phases during stepping (Phase 1: 360; Phase 2: 720; Phase 3: 1080; Phase 4: 1440; Phase 5: 1800; Phase 6: 2160 ms after maximum extension of the left hip joint). Phases 1–3 represent the stance phase of the right leg and Phases 4–6 represent the swing phase of the right leg. Each stimulus was delivered at intervals of > 8 s. First, the maximum motor response (Mmax) amplitudes were measured during passive standing. Based on the Mmax value, the stimulus intensity was adjusted to approximately 10% of Mmax. Second, 15 H-reflexes were measured during passive standing. Third, 15 H-reflexes were recorded during each stepping phase. As a practice for each stepping task, the participants practiced for approximately five minutes before starting the measurement. The order of voluntary and passive stepping was randomized for each participant. Finally, EMG signals were recorded during MVC.

### Electromyographic (EMG) recording

Surface EMG signals were recorded using Ag/AgCl electrodes (F-150S; Nihon Kohden, Tokyo, Japan). Surface electrodes were placed over the muscle bellies of the right FCR, extensor carpi radialis (ECR), rectus femoris (RF), biceps femoris (BF), tibialis anterior (TA), and soleus (SOL) after the skin was cleaned using abrasion and alcohol wipes. Due to the limited number of electrodes in the EMG, the left leg muscles were not measured in these experiments. EMG signals were amplified 1000 times and band-pass filtered (15 Hz–3 kHz) using a bioamplifier system (MEG-6108; Nihon Kohden, Tokyo, Japan).

### Data recording and analysis

The analog signals of the EMG, as well as the angles of the hip and knee joints, were converted into digital data at 5 kHz using an A/D converter (Micro1401; CED Ltd., Cambridge England, UK) and stored on a hard disk. Data were then analyzed offline using a custom-written MATLAB program (MathWorks Inc., Natick, Massachusetts, USA). TMS coil positions at each stimulation were obtained using the LabVIEW program (National Instruments, Texas, USA).

The peak-to-peak amplitudes of the MEPs were averaged for each phase of each stepping condition, as well as for the standing condition. The stepping condition amplitudes were normalized to the amplitudes of the standing condition. By using this normalization method, it is possible to include information about the MEP in the standing condition for each subject in the MEP values for the stepping condition. The peak-to-peak amplitudes of the H-reflex and M-wave were averaged for each phase of each stepping condition, as well as for the standing condition. These amplitudes were normalized to the maximum amplitude obtained in the standing condition. The stepping condition amplitudes were normalized to the standing condition amplitudes. The background EMGs of the arm and leg muscles were calculated using the root-mean-square (RMS) for 50 ms before TMS or electrical stimulation for the H-reflex recording and these RMS values were normalized with RMS values of EMG during MVC.

### Statistical analysis

In Experiments 1 and 2, one-way repeated measures analysis of variance (RM-ANOVA) was applied to the normalized MEP amplitudes and background EMGs of the six stepping phases and passive standing. When a significant main effect was detected using one-way RM-ANOVA, Bonferroni’s test for multiple comparisons was performed.

In Experiment 3, one-way repeated measures analysis of variance (RM-ANOVA) was applied to the normalized H-reflexes, normalized M-waves, and background EMGs of the six stepping phases and passive standing. When a significant main effect was detected using one-way RM-ANOVA, Bonferroni’s test for multiple comparisons was performed. The voluntary stepping and passive stepping conditions were analyzed separately.

One-way RM-ANOVA is a statistical test that assumes normality. Shapiro–Wilk test was used to evaluate normal distribution of data. If the data were not normally distributed, the Friedman repeated measures analysis of variance on ranks was used instead of one-way RM-ANOVA. Tukey’s test was used as a test for the multiple comparisons.

All statistical tests were performed using SigmaPlot 14 (Systat Software, Inc., San Jose, CA, USA). Using this software, a power test was performed simultaneously with a one-way ANOVA. Those with a power below 80% are described in the results. Statistical significance was set at *p* < 0.05.

## Results

### MEP amplitudes and background EMG during voluntary stepping (experiment 1)

Figure 2B shows the typical MEP data during voluntary stepping. The MEP amplitudes were larger during voluntary stepping than during standing (Fig. 2B). One-way RM-ANOVA showed a statistically significant main effect for voluntary stepping phases and passive standing (Fig. 2C; F _(6, 48)_ = 7.027, *p* < 0.001). The Bonferroni multiple comparison test revealed significant differences among phases (Fig. 2C *p* < 0.05). In addition, the MEPs of Phases 2, 3, and 6 were significantly larger than those of passive standing (Fig. 2C, *p* = 0.002, *p* = 0.005, and *p* < 0.001, respectively).

**Fig. 3:**
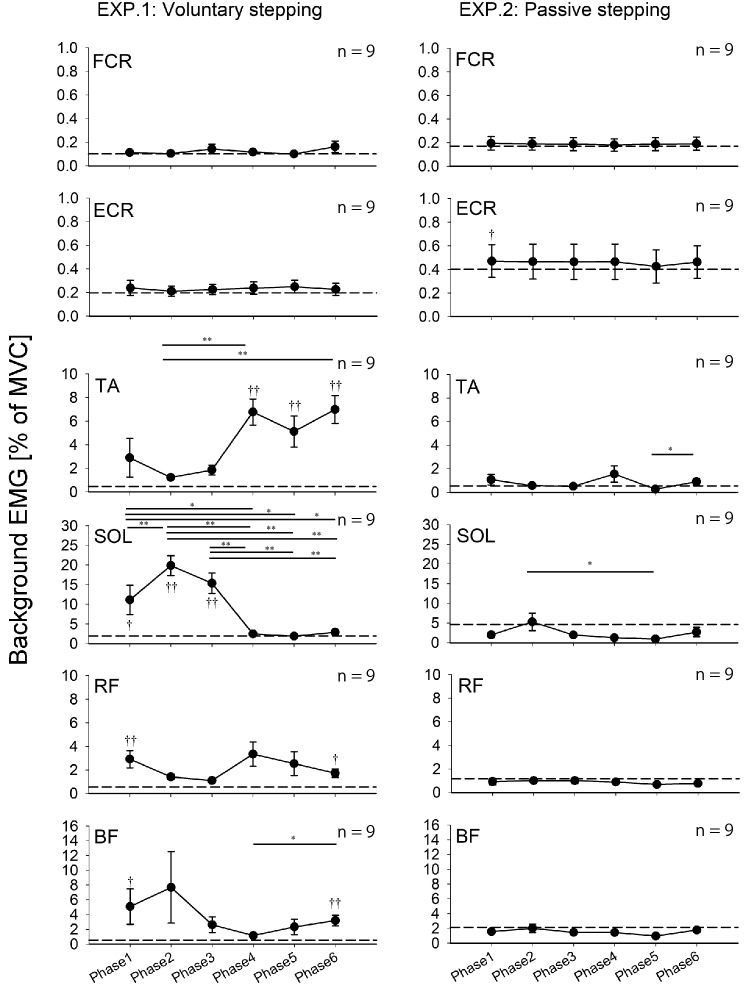
Average data (± standard error) of the root-mean-squares of background EMG activities obtained from nine subjects during voluntary and passive stepping. These data were recorded in Experiments 1 and 2 (TMS experiments). The filled circles indicate the average background activity values at six different phases of voluntary and passive stepping, respectively. Dotted lines indicate the average values of background activities during standing (*p < 0.05; **p < 0.01 for difference between the stepping phases; † *p* < 0.05; ††*p* < 0.01 for difference between each stepping phase and passive standing). Abbreviations: *BF* biceps femoris, *ECR* extensor carpi radialis, *EMG* electromyography, *FCR* flexor carpi radialis, *MVC* maximum voluntary contraction, *RF* rectus femoris, *SOL* soleus, *TA* tibialis anterior, *TMS* transcranial magnetic stimulation

The results of background EMG recordings during voluntary stepping are shown in Fig. 3. For FCR and ECR muscles, a Friedman test revealed no significant main effect for voluntary stepping phases and passive standing (FCR: χ^2^ (6) = 2.714, *p* = 0.844, ECR: χ^2^ (6) = 9.000, *p* = 0.174). For TA, RF, and BF muscles, a Friedman test showed a statistically significant main effect for voluntary stepping phases and passive standing (TA: χ^2^ (6) = 38.952, *p* < 0.001; RF: χ^2^ (6) = 19.667, *p* = 0.003; BF: χ^2^ (6) = 30.00, p < 0.001). For the SOL muscle, one-way RM-ANOVA showed a statistically significant main effect for voluntary stepping phases and passive standing (F _(6, 48)_ = 17.594, *p* < 0.001). The results obtained from all pairwise multiple comparison procedures are shown in Fig. 3.

### MEP amplitudes and background EMG during passive stepping (experiment 2)

Figure 2E shows typical MEP data during passive stepping. The MEP amplitudes during passive stepping were as large as those during standing (Fig. 2E). A Friedman test revealed no significant main effect for passive stepping phases and passive standing (Fig. 2F; χ^2^ (6) = 3.381, *p* = 0.760).

The results of background EMG during passive stepping are shown in Fig. 3. For the FCR muscle, Friedman repeated measures analysis of variance on ranks revealed no significant main effect for passive stepping phases and passive standing (χ^2^ (6) = 11.619, *p* = 0.071). For BF muscle, one-way RM-ANOVA revealed no significant main effect for passive stepping phases and passive standing (F _(6, 48)_ = 1.936, *p* = 0.094). However, the power of the test (0.333) was below the desired power of 0.800. For ECR, TA, SOL, and RF muscles, Friedman repeated measures analysis of variance on ranks revealed statistically significant main effects for passive stepping phases and passive standing (ECR: χ^2^ (6) = 16.095, *p* = 0.013, TA: χ^2^ (6) = 38.952, *p* < 0.001; SOL: χ^2^ (6) = 14.048, *p* = 0.029; RF: χ^2^ (6) = 12.714, *p* = 0.048). The results obtained from all pairwise multiple comparison procedures are shown in Fig. 3.

### H-reflex and M-wave amplitudes during voluntary and passive stepping (Experiment 3)

**Fig. 4:**
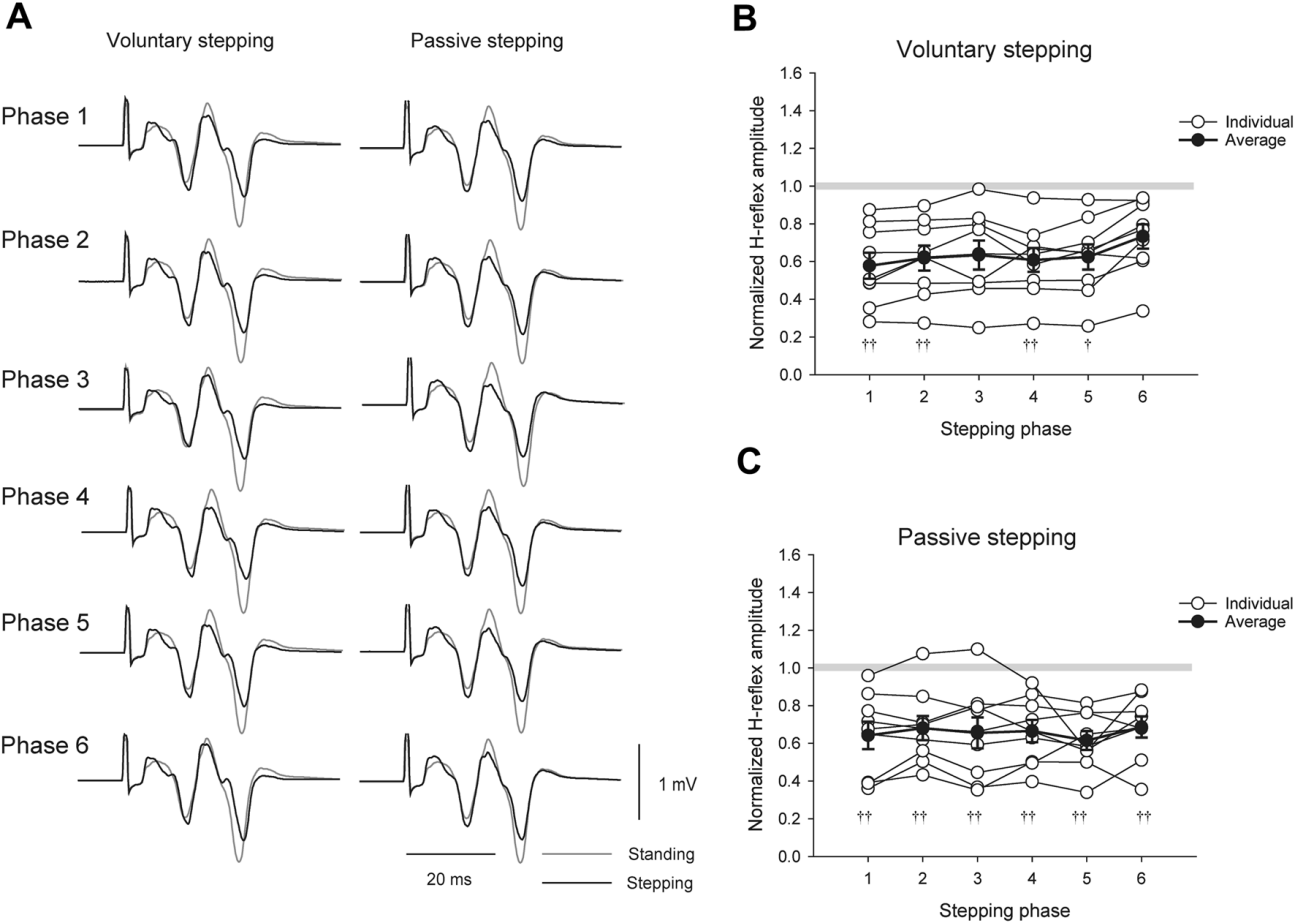
H-reflex responses obtained during voluntary and passive stepping tasks. Black lines indicate typical H-reflex waveforms (15 sweeps) obtained from FCR muscle during passive and during voluntary stepping (**A**). Gray lines indicate the H-reflex responses recorded during standing. (**B**) and (**C**) Individual data (unfilled circles) and average data (filled circles), with the standard error of the H-reflex amplitudes in the FCR muscle obtained from nine subjects during voluntary (**B**) and passive stepping (**C**), respectively. The H-reflex amplitudes were normalized to the H-reflex amplitudes of the standing condition (†*p* < 0.05; ††*p* < 0.01 for difference between each stepping phase and passive standing). Abbreviations: *FCR* flexor carpi radialis, *H-reflex* Hoffmann reflex

Figure 4A shows the typical data of the H-reflexes during voluntary and passive stepping. The amplitudes of the H-reflexes were smaller during both stepping conditions than during standing. For voluntary stepping condition, a Friedman repeated measures test revealed a significant main effect for stepping phases and passive standing (Fig. 4B; χ^2^ (6) = 32.095, *p* < 0.001). Tukey’s multiple comparison test revealed significant differences between Phases 1 and 6 (Fig. 4B, *p* < 0.05). In addition, the H-reflexes of Phases 1, 2, 4, and 5 of voluntary stepping were smaller than those of passive standing (*p* < 0.001, *p* < 0.001, *p* = 0.004, and *p* = 0.026, respectively). For the passive stepping condition, one-way RM-ANOVA showed a statistically significant main effect for stepping phases and passive standing (Fig. 4C; F _(6, 48)_ = 12.695, *p* < 0.001). A Bonferroni multiple comparison test revealed that H-reflexes of Phases 1, 2, 3, 4, 5, and 6 were smaller than those of passive standing (*p* < 0.001).

For the normalized M-wave of voluntary stepping condition, one-way RM-ANOVA showed no significant main effect for stepping phases and passive standing (F _(6, 48)_ = 1.095, *p* = 0.379). However, the power of the test (0.071) was below the desired power of 0.800. For the normalized M-wave of the passive stepping condition, a Friedman test revealed no significant main effect for stepping phases and passive standing (χ^2^ (6) = 8.190, *p* = 0.224).

**Fig. 5:**
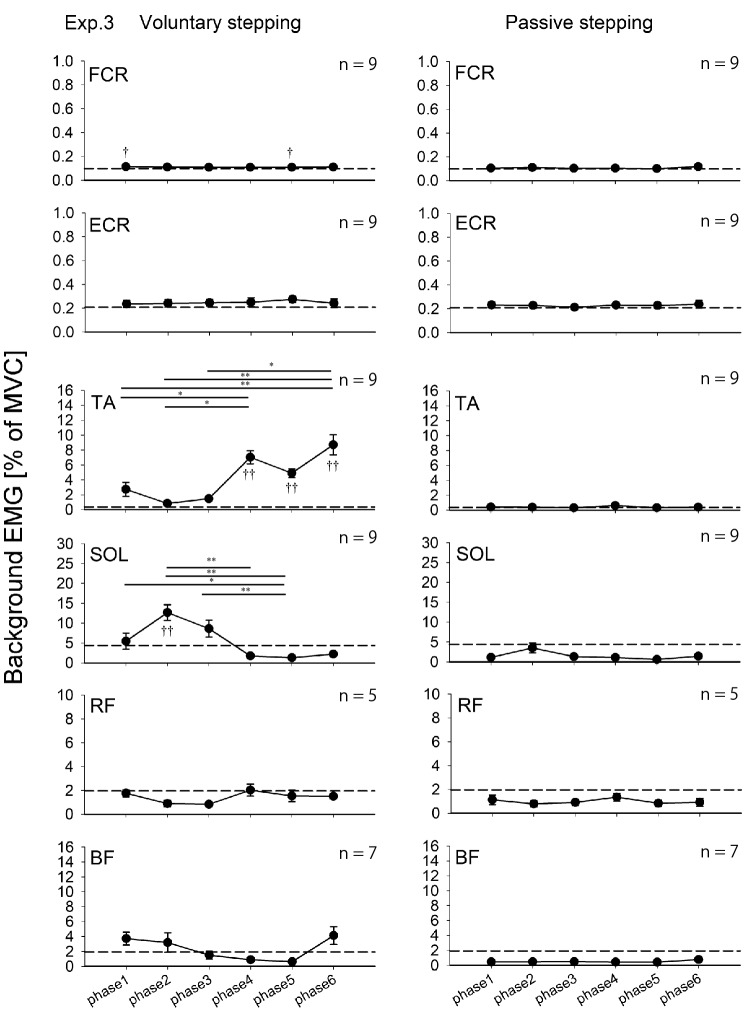
Average (± standard error) of the root-mean-squares of background EMG activities obtained from the subjects during voluntary and passive stepping. These data were recorded in Experiment 3 (H-reflex experiment). The filled circles indicate the average background activity values at six different phases of voluntary and passive stepping, respectively. Dotted lines indicate the average values of background activities during standing. (*p < 0.05; **p < 0.01 for difference between the stepping phases; †*p* < 0.05;†† *p* < 0.01 for difference between each stepping phase and passive standing). Abbreviations: *BF* biceps femoris, *ECR* extensor carpi radialis, *EMG* electromyography, *FCR* flexor carpi radialis, *MVC* maximum voluntary contraction, *RF*: rectus femoris, *SOL* soleus, *TA* tibialis anterior, *H-reflex* Hoffmann reflex

Background EMG data were analyzed for each muscle during each stepping condition. For voluntary stepping condition, a Friedman rank test revealed statistically significant main effects for stepping phases and passive standing in background EMG of FCR, TA, and SOL (FCR: χ^2^ (6) = 14.714, *p* = 0.023, TA: χ^2^ (6) = 48.095, *p* < 0.001; SOL: χ^2^ (6) = 35.524, *p* < 0.001), while a Friedman rank test revealed no significant main effect for stepping phases and passive standing in background EMG of ECR (χ^2^ (6) = 9.000, *p* = 0.174). For the passive stepping condition, a Friedman rank test revealed statistically significant main effects for stepping phases and passive standing in background EMG of SOL (χ^2^ (6) = 14.143, *p* = 0.028), while a Friedman rank test revealed no significant main effect for stepping phases and passive standing in background EMG of FCR, ECR, and TA (FCR: χ^2^ (6) = 12.190, *p* = 0.058, ECR: χ^2^ (6) = 2.857, *p* = 0.827, TA: χ^2^ (6) = 6.905, *p* = 0.330). In Experiment 3, the EMG electrodes were in contact with the cuff of Lokomat, which prevented us from recording the EMG activity of the RF and BF muscles of several subjects, so we did not perform statistical analysis of the background EMG activity of the RF and BF muscles. The results obtained from all pairwise multiple comparison procedures are shown in Fig. 5.

## Discussion

The present study investigated excitability in the corticospinal tract and the monosynaptic reflex circuits of the forearm muscle during passive and voluntary stepping. The results showed that the MEP of the FCR was facilitated phase-dependently during voluntary stepping, while no such modulation was observed during passive stepping. Regarding the monosynaptic reflex, the H-reflexes were suppressed during both voluntary and passive stepping, as compared to passive standing. These results suggest that (1) voluntary commands to leg muscles, combined with somatosensory inputs during walking, facilitate the corticospinal excitability of forearm muscles in a phase-dependent manner and (2) somatosensory inputs during walking suppress the excitability of the monosynaptic reflex in the forearm muscle.

### Effects of stepping-induced afferent information on corticospinal excitability in forearm muscle

The passive movement paradigm has been used to investigate the effects of afferent information on the central nervous system (Brooke et al. 1995, 1997 a & b; Kamibayashi et al. 2010; Nakajima et al. 2011). In this paradigm, to eliminate the influence of descending commands, participants were instructed to relax their whole body during passive stepping. As a result, the background EMGs of the arm and leg muscles were small, although the SOL muscles were involuntarily activated during the stance phase. In addition, the movement of robot-assisted stepping is very similar to that of walking, with standing posture, foot contact, and joint movement. In the present study, all participants were able to perform the passive stepping task, and the results of this task likely reflected the effects of afferent information on MEP amplitudes in the forearm muscle during walking.

The amplitude of the MEP in single-pulse TMS is thought to be a measure of motoneuron excitability in the corticospinal pathway from the primary motor cortex to the targeted muscle. Using the single-pulse TMS technique, we examined corticospinal excitability in the leg muscles during passive stepping in our previous study (Kamibayashi et al. 2010), which showed that the MEP amplitude of the ankle dorsiflexor muscle (e.g., TA muscle) increased during passive ground stepping, but not during passive air stepping, as compared with passive standing. This suggests that load-related sensory inputs during walking facilitate corticospinal excitability in the leg muscles. In contrast, in the present study, the MEP amplitude of the FCR muscle (forearm muscle) was not modulated by passive ground stepping. Although not all muscles in both the arm and leg were examined, it may be that afferent information during walking has different neural effects on corticospinal excitability between the arm and leg muscles.

Passive cycling and single joint movement have often been used to examine the effects of afferent information on the central nervous system. However, to our knowledge, no studies have addressed the effects of locomotor-related afferent inputs on corticospinal excitability in forearm muscles. The present results showed, for the first time, that movement- and load-related afferents had no statistically significant effect on the corticospinal excitability of the forearm muscle during walking.

### Effects of voluntary commands to leg muscles on corticospinal excitability in the forearm muscle

The present study showed that the MEP of the FCR muscles was modulated phase-dependently during voluntary stepping. The background EMG activity of the forearm muscle was negligible and remained unchanged during voluntary stepping, indicating that the modulation of MEP amplitude was not caused by background EMG activities of the forearm muscle during voluntary stepping. The MEP amplitude is thought to be modulated by both supraspinal and spinal inputs; therefore, the present study cannot conclude the detailed neural mechanisms. However, based on previous studies, the present results provide some insight into the mechanisms underlying the increased MEP amplitude during voluntary stepping.

There is abundant evidence of neural interactions between the arm and leg muscles (Péréon et al. 1995; Delwaide and Toulouse 1981). A previous study showed that leg muscle contraction enhances corticospinal tract excitability in the FCR muscle (Tazoe et al. 2007). This facilitation was induced by strong muscle activity, which was greater than 50% of MVC. However, in the present study, the maximum values of TA and SOL muscle activity during voluntary stepping were very small (TA: 6.99% ± 1.34% of MVC, SOL: 19.84% ± 2.86% of MVC). In addition, previous studies have shown that remote muscle contraction enhances both corticospinal excitability (Sugawara et al. 2002; Tazoe et al. 2007) and monosynaptic reflex excitability (Miyahara et al. 1996; Sugawara et al., 2002). However, the present results showed strong suppression of the H-reflex in the forearm muscle during voluntary stepping. Therefore, the present results cannot be explained in terms of remote facilitatory effects. Voluntary commands for leg stepping combined with locomotor-related afferent inputs may cause strong MEP facilitation. These results were consistent with a previous study showing the neural effects of voluntary leg cycling movement on corticospinal excitability in the forearm muscle. Zehr et al. (2007) showed that rhythmic leg cycling facilitates corticospinal excitability in forearm muscles. They also investigated the conditioning effects of subthreshold TMS on the size of the forearm H-reflex. The subthreshold TMS conditioning effects were similar during leg cycling and at rest. Therefore, they concluded that subcortical activity is responsible for increased corticospinal excitability during leg cycling. Taking these results into consideration, voluntary stepping may modulate forearm corticospinal tract excitability at the subcortical level.

### Effects of stepping on monosynaptic reflex excitability in forearm muscle

In addition to corticospinal excitability, the present study assessed excitability in the monosynaptic reflex of the forearm muscle during voluntary and passive stepping. Although the results showed no difference between voluntary and passive stepping conditions, the H-reflex amplitude was strongly suppressed during both voluntary and passive stepping, as compared with passive standing. This result is consistent with previous studies (Nakajima et al. 2011; Domingo et al. 2014) and suggests that movement-related sensory inputs induced by stepping play an important role in H-reflex suppression. In addition, because the experimental setting did not exclude elbow and shoulder movements, sensory input from the arms as well as the legs may partially involve in the H-reflex suppression. It was suggested that somatosensory inputs from the legs during walking may enhance presynaptic inhibition of Ia terminals in the monosynaptic reflex circuits of forearm muscles (Frigon et al. 2004; Nakajima et al. 2016). Moreover, presynaptic inhibition is thought to reduce the effectiveness of afferent inputs generated by movement (Seki et al. 2003). Therefore, somatosensory input during walking may allow voluntary commands to activate the forearm muscles easily.

### Functional implication

Several studies have shown that the EMGs of the shoulder (posterior deltoid, middle deltoid, and anterior deltoid) and upper-arm muscles (biceps brachii and triceps brachii) are enhanced in late-to-terminal stance and late-to-terminal swing (Ballesteros et al. 1965; Kuhtz-Buschbeck and Jing, 2012). Interestingly, these EMG patterns were similar to the phase-dependent modulation of MEP amplitudes in the present study. That is, the MEP was especially enhanced in the late-to-terminal stance and terminal-swing phase during voluntary stepping. Barthelemy and Nielsen (2010) revealed that the primary motor cortex contributes to muscle activation during arm swings. It follows that the changes in corticospinal excitability shown in this study are associated with the arm muscle activation pattern during normal walking. In this study, the subjects were instructed to relax the muscles of arms, and no EMG signals were observed in the arm muscles. This suggests that the interlimb neural mechanism of walking is unconsciously driven by descending commands to the legs combined with sensory information.

### Limitations

This study had some limitations. First, it is difficult to directly compare and discuss the MEP data of voluntary and passive stepping because the measurements were performed on different subjects over two days. The difference between passive and voluntary stepping should be investigated in detail in the future. Second, in this study, EMGs were obtained from the right leg and right forearm muscles. Due to the limited number of electrodes in the EMG, we were not able to measure the activity of other muscles, such as the contralateral leg muscles and the upper-arm and shoulder muscles. The effect of the activity of these muscles on the results of this study is unknown. Third, kinematic and kinetic data from the arm were not recorded during either voluntary or passive stepping. Therefore, we cannot exclude the possibility that differences in arm motion between stepping conditions contributed to the differences in MEP regulation that we observed. It is well known that the afferent feedback from muscles spindles, joints and cutaneous fields modulate H-reflex (Agostinucci et al. 2006; Baldissera et al. 2000; Brooke et al. 1995, 1997; Masugi et al. 2015; Nakajima et al. 2012; Kamibayashi et al. 2010) and MEPs (Ginanneschi et al. 2006; Kamibayashi et al.^2009^; Mitsuhashi et al. 2007). However, in our study the FCR H-reflex showed no differences in regulation between the voluntary and passive stepping conditions, suggesting that any differences in arm motion that might have occurred did not greatly influence this reflex arc. Therefore, it is also unlikely that differences in afferent input from the arm was major factor related to the differences in MEP regulation we observed between voluntary and passive stepping. Moreover, previous studies showed that the MEP amplitudes in the resting FCR muscle were changed by voluntary command to other parts of limb (Baldissera et al. 2002; Tazoe et al. 2007, 2009), consistent with our observation that voluntary activation of leg muscles during stepping also influences MEP amplitudes. Fourth, to prevent involuntary muscle activity and motion artifacts, the walking speed of the task in this experiment was set as slow. It is possible that walking at this slower speed affects the voluntary control of walking. These effects must be considered when interpreting the results of this study.

## Conclusion

Voluntary commands to the leg muscles, combined with somatosensory inputs, may facilitate corticospinal excitability in the forearm muscle, while somatosensory inputs during walking play a role in the suppression of monosynaptic reflex excitability in the forearm muscle. The results of this study are important for the elucidation of neural control mechanisms in the arms and legs during human walking.

## Data Availability

The datasets generated and/or analyzed during the current study are available from the corresponding author upon reasonable request.
